# Expression of Concern: α-bisabolol enhances radiotherapy-induced apoptosis in endometrial cancer cells by reducing the effect of XIAP on inhibiting caspase-3

**DOI:** 10.1042/BSR-20190696_EOC

**Published:** 2020-07-02

**Keywords:** endometrial cancer, α-bisabolol, radiotherapy, apoptosis, XIAP/caspase-3 pathway

The Editorial Office has been made aware of potential issues surrounding the scientific validity of this paper, hence has issued an expression of concern to notify readers whilst the Editorial Office investigates.

